# Atomic Pathways of Ammonia-Driven Fe_3_O_4_ Reduction Revealed by First-Principles Calculations

**DOI:** 10.1021/acs.jpcc.6c01024

**Published:** 2026-02-27

**Authors:** Zhikang Zhou, Linna Qiao, Shuonan Ye, Mengen Wang, Guangwen Zhou

**Affiliations:** † Department of Mechanical Engineering & Materials Science and Engineering Program, Binghamton University, State University of New York, Binghamton, New York 13902, United States; ‡ Department of Physics and Astronomy, University of North Carolina at Chapel Hill, Chapel Hill, North Carolina 27599, United States

## Abstract

The direct reduction of iron ore using hydrogen faces challenges associated with hydrogen storage, transport, and on-site handling. Ammonia (NH_3_), with its high hydrogen content, established distribution infrastructure, and economic viability, has emerged as a promising alternative reductant. Here, we employ density functional theory calculations to elucidate the atomic-scale mechanisms governing NH_3_ adsorption, dehydrogenation, and nitrogen incorporation on the Fe_3_O_4_(001) surface. Our results show that NH_3_ preferentially adsorbs upright at the surface Fe sites, initiating a sequence of dehydrogenation steps. Among the three dehydrogenation reaction pathways examined, H migration is identified as the rate-determining step for H_2_O formation and desorption, a process that generates surface oxygen vacancies. The resulting NH and N species strongly bind to the surface through multiple Fe–N and Fe–NH coordination bonds. Notably, the most favorable configuration—NH binds adjacent to an oxygen vacancy—facilitates further NH dissociation into N and H. The generated vacancies migrate favorably into the subsurface, enabling N incorporation into the lattice and promoting the formation of Fe nitride. Concurrently, N atoms that do not incorporate recombine to form N_2_, thereby preventing excessive N accumulation on the surface. These results provide atomistic insights into NH_3_-driven Fe_3_O_4_ reduction and reveal the coupled vacancy dynamics, H mobility, and N incorporation pathways that underpin NH_3_-based ironmaking, highlighting the mechanistic opportunities for optimizing sustainable iron ore reduction and advancing NH_3_-enabled catalytic processes.

## Graphical abstract

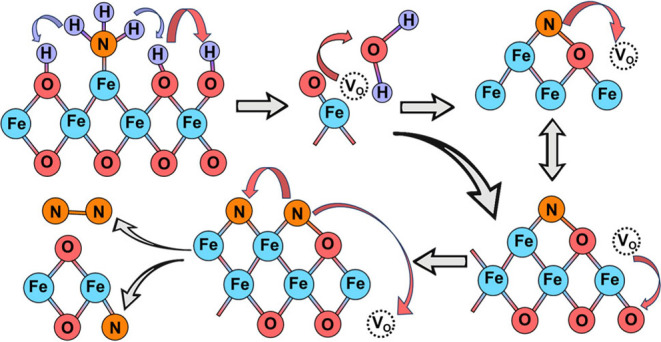

## 1 Introduction

Iron and steel production remains a cornerstone of global industry but is also responsible for nearly 7% of anthropogenic CO_2_ emissions, largely due to its dependence on carbon-based reductants such as coal, coke, and carbon monoxide.^1–5^ The pursuit of low-carbon steelmaking has therefore driven interest in hydrogen-based direct reduction, which generates only H_2_O and can be paired with renewable-powered electrolysis to enable circular operation. Realizing this vision, however, requires a deep understanding of how hydrogen interacts with iron oxides at the atomic scale. Prior theoretical and experimental studies have elucidated key aspects of H_2_ adsorption, dissociation, and H_2_O formation^6–12^ as well as reduction kinetics and phase transformation pathways.^13–20^

Despite its advantages as a clean reductant, widespread adoption of H_2_ is constrained by storage, transport, and infrastructure challenges, owing to its low volumetric energy density (∼3 kWh/m^3^) and the need for energy-intensive compression or liquefaction.^21–24^ Additionally, the small molecular size and high diffusivity of H_2_ make it prone to leakage, increasing safety risks during transport. These limitations have motivated a strong interest in ammonia (NH_3_) as an alternative hydrogen carrier for iron one reduction.^25–27^ NH_3_ offers higher volumetric energy density, is easily liquidized (−33 °C at 1 atm),^28^ and benefits from mature global handling infrastructure. Moreover, NH_3_ contains 17.75 wt % hydrogen and can be synthesized using renewable H_2_, enabling a fully carbon-free supply chain.^28^

In ironmaking environments, NH_3_ dehydrogenates catalytically, yielding reactive H species in situ while releasing N_2_.^29,30^ N incorporation into partially reduced iron can also form a protective nitride phase, enhancing downstream stability.^31^ Although NH_3_-oxide interactions have been explored on TiO_2_ and selected Fe oxide surfaces,^32–35^ results remain system-specific and often show competing effects: NH_3_ can exhibit strong adsorption, altered dissociation behavior in the presence of defects or dopants, and varying reduction efficiencies depending on temperature and surface structure. Experimental comparisons of NH_3_- and H_2_-based reduction further highlight the complex interplay between NH_3_ chemistry, reduction kinetics, and nitride formation.^25–27^

Despite this progress, the atomistic mechanisms governing the early stages of NH_3_ reduction of iron oxides remain poorly resolved. Fundamental questions persist regarding NH_3_ adsorption geometries, dehydrogenation pathways, H migration, H_2_O formation, N incorporation, and vacancy evolution—processes that ultimately dictate reduction efficiency and product phases. To address these gaps, we investigate NH_3_ reaction chemistry on the Fe_3_O_4_(001) surface, a well-characterized and technologically important iron oxide relevant to H_2_- and NH_3_-based direct reduction. Although the Fe_3_O_4_(001) surface has been extensively studied under H_2_ environments,^36,37^ its behavior under NH_3_ remains largely unexplored. Given the critical global push toward decarbonization of the steel industry, there is an urgent need for quantitative, mechanistic models that can complement and guide ongoing experimental efforts. Using density-functional theory (DFT), we elucidate the detailed mechanisms of NH_3_ adsorption, H migration, H_2_O formation, N evolution, and vacancy formation. These insights advance the atomic-level understanding of NH_3_-driven Fe oxide reduction and provide guidance for designing efficient NH_3_-enabled pathways for sustainable ironmaking.

## 2 Computational Methods

The Fe_3_O_4_(001) surface, one of the most common facets of magnetite, consists of alternating A-layers (tetrahedrally coordinated Fe^3+^) and B-layers (octahedrally coordinated Fe^2+^/Fe^3+^). Prior studies have established that the B-terminated configuration is energetically favored,^38–40^ and this surface undergoes a characteristic √2 × √2*R*45° reconstruction observed experimentally.^41,42^ We adopt a √2 × √2*R*45° B-terminated Fe_3_O_4_(001) slab model with in-plane dimensions *a* = *b* = 11.95 Å and a slab thickness of *c* = 23.54 Å, separated by a 15 Å vacuum region to avoid spurious interslab interactions. This supercell size is specifically chosen to maintain the spatial isolation of oxygen vacancies (V_O_) generated during the H_2_O desorption process, thereby minimizing artificial defect–defect interactions between periodic images. We note that the adsorption of basic molecules may induce the “lifting” of the √2 × √2*R*45° reconstruction toward a bulk-terminated (1 × 1) state, as has been reported for other adsorbates.^43–45^ While such a global phase transition can influence absolute adsorption enthalpies and long-range vacancy diffusion kinetics, the current study focuses on the local elementary steps of NH_3_ activation. Because these pathways are primarily dictated by the immediate coordination environment and the electronic structure of the active site, the mechanistic trends reported here—particularly the competition between proton transfer and concerted elimination—are expected to be representative of the local defect chemistry regardless of the long-range surface symmetry.

All DFT calculations are performed using the Vienna Ab initio Simulation Package (VASP), within the Kohn–Sham formalism and projector-augmented wave (PAW) pseudopotentials.^46–49^ Exchange-correlation effects are described using the PBE functional,^50^ and strong on-site Coulombic interactions of Fe 3d electrons are teated within the DFT + *U* framework,^51^ employing *U* = 3.8 eV in accordance with previous studies of Fe_3_O_4_.^51,52^ A plane-wave cutoff of 520 eV is used. Structural optimization adopts a force convergence threshold of 0.02 eV/Å. Brillouin zone sampling employs a 3 × 3 × 1 Γ-centered *k*-point mesh. All calculations are spin-polarized, with initial magnetic moments assigned according to the ferrimagnetic ordering of Fe_3_O_4_. NH_3_ adsorption energies are calculated using *E*_ad_ = *E*_NH_3_/surface_ – *E*_surface_ – *E*_NH_3__, where the terms correspond to the energies of the adsorbed system, the pristine surface, and an isolated NH_3_ molecule, respectively. Reaction energy barriers for key elemental steps, including NH_3_ dissociation and H migration, are determined using the *Climbing Image Nudged Elastic Band* (CINEB) method^53^ to locate transition states.

## 3 Results and Discussion

### 3.1 NH_3_ Adsorption

We begin by examining molecular NH_3_ adsorption on Fe_3_O_4_(001). Figure 1a shows the top view of the relaxed pristine surface and identifies four symmetry-distinct adsorption sites for NH_3_: (1) atop a surface Fe cation, (2) a hollow site between two Fe cations, (3) atop a surface O anion with the H oriented toward O, (3′) atop a surface O anion with the N lone pair oriented toward O, and (4) atop a subsurface Fe cation. Structural relaxation reveals that configurations initiated at sites 1–3 all converge to the same adsorption geometry (Figure 1b), in which the NH_3_ binds through its N atom to a surface Fe cation. Site 3′ relaxes to a different local minimum (Figure 1c), and adsorption at site 4 yields a distinct configuration (Figure 1d).

**Figure 1:**
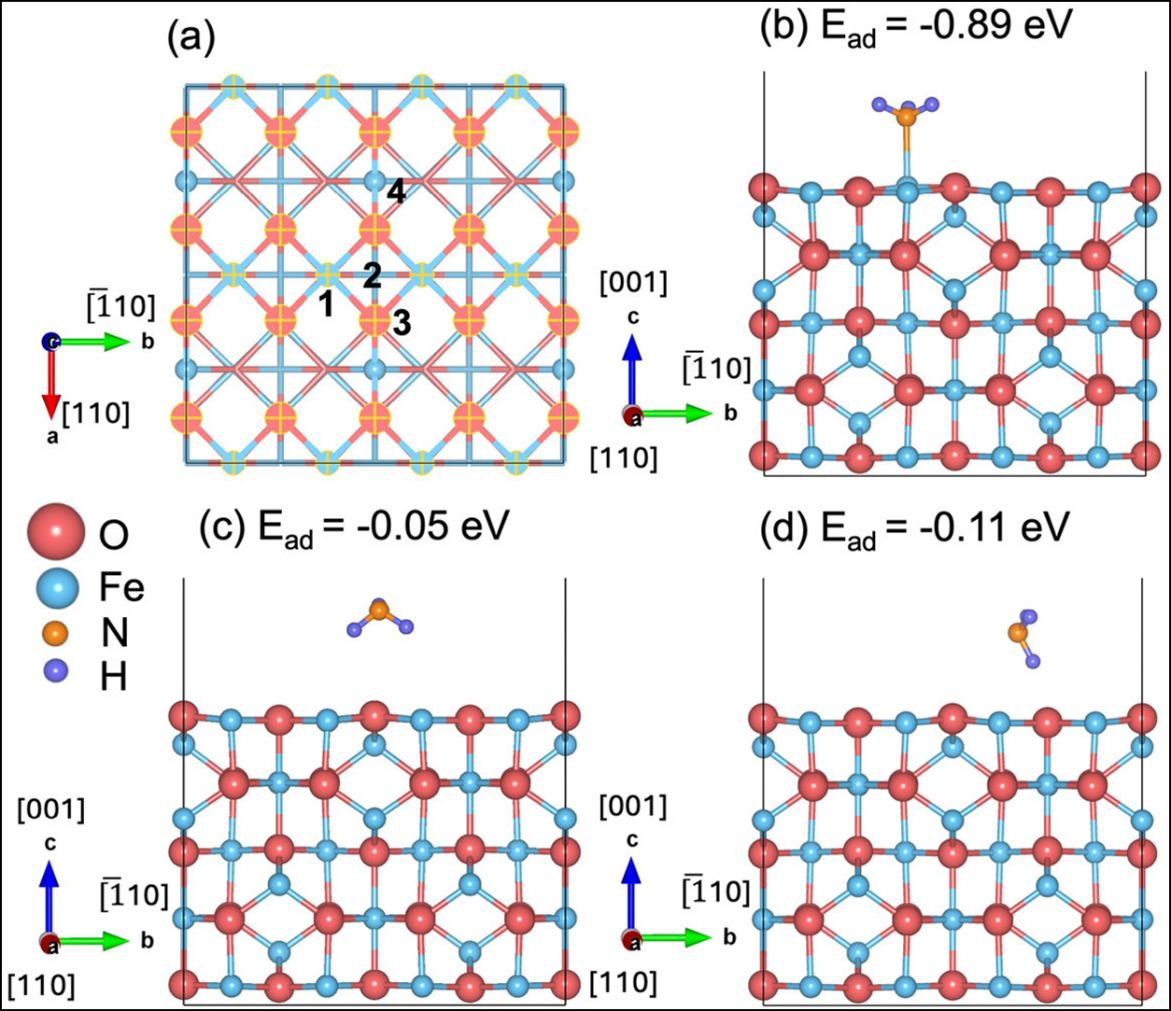
Adsorption behavior of NH_3_ on the Fe_3_O_4_(001) surface. NH_3_ preferentially binds at surface Fe sites in an upright orientation, forming a strong N–Fe bond in the most stable adsorption geometry. (a) Top view of the √2 × √2*R*45° Fe_3_O_4_(001) slab, showing four symmetry-distinct adsorption sites. Only the top two atomic layers are shown, with the first layer highlighted for clarity. (b–d) Relaxed adsorption configurations and their corresponding energies: (b) the most stable geometry, with NH_3_ upright and coordinated through N to a surface Fe cation; (c) a metastable local minimum with NH_3_ interacting with a surface O site; (d) adsorption above a subsurface Fe cation, yielding a distinct configuration.

Among all optimized structures, the configuration in Figure 1b is the most stable, exhibiting an adsorption energy of −0.89 eV. This reflects a strong interaction between the NH_3_ lone-pair orbital toward the surface Fe cation. The resulting N–Fe bond length of 2.197 Å agrees well with previously reported (2.125–2.319).^34,35,54,55^ The preference for N-down binding is consistent with NH_3_ acting as a Lewis base: its nonbonding lone pair, originating from the highest occupied molecular orbital (HOMO) with the valence electron configuration of (1a_1_)^2^(1e)^2^(1e)^2^(2a_1_)^2^ under *C*_3*v*_ symmetry,^56^ readily donates electron density to the surface. Bader charge analysis confirms this donation, showing a charge transfer of ∼0.13 e from NH_3_ to the Fe_3_O_4_ in the optimized structure. Given its clear energetic preference and electronic characteristics, the adsorption geometry in Figure 1b is selected as the initial state for probing subsequent NH_3_ dehydrogenation and Fe_3_O_4_ reduction pathways.

### 3.2 NH_3_ Dissociation

As shown in Figure 2, we identify three possible pathways for NH_3_ oxidative dehydrogenation on Fe_3_O_4_(001) and determine the minimum-energy configurations associated with each step. The reaction begins with NH_3_ adsorbed upright on a surface Fe site, forming a stable precursor state with an adsorption energy of −0.89 eV. Activation of NH_3_ proceeds via a two-step sequence: (1) lateral translation of the adsorbed NH_3_ molecule along the [110] direction, followed by (2) rotation of an N–H bond toward the [001̅] direction to align for cleavage. This motion brings NH_3_ into the transition-state geometry required for the first dehydrogenation step. The initial N–H scission step requires an energy barrier of 1.19 eV, in excellent agreement with previously reported values (1.20 eV).^34^ During this process, one H atom detaches from NH_3_ and binds to a nearby lattice O atom, forming a surface OH group. After dissociation, the OH species tilts slightly along [110], while the remaining NH_2_ fragment relaxes back to an upright configuration, yielding a stable intermediate denoted as (NH_2_ + OH)*. Thermodynamically, this first dehydrogenation step is exothermic by 0.43 eV, demonstrating that once NH_3_ adsorption occurs, the Fe_3_O_4_(001) surface provides a favorable energetic landscape for NH_3_ activation and initiation of the reduction process.

**Figure 2:**
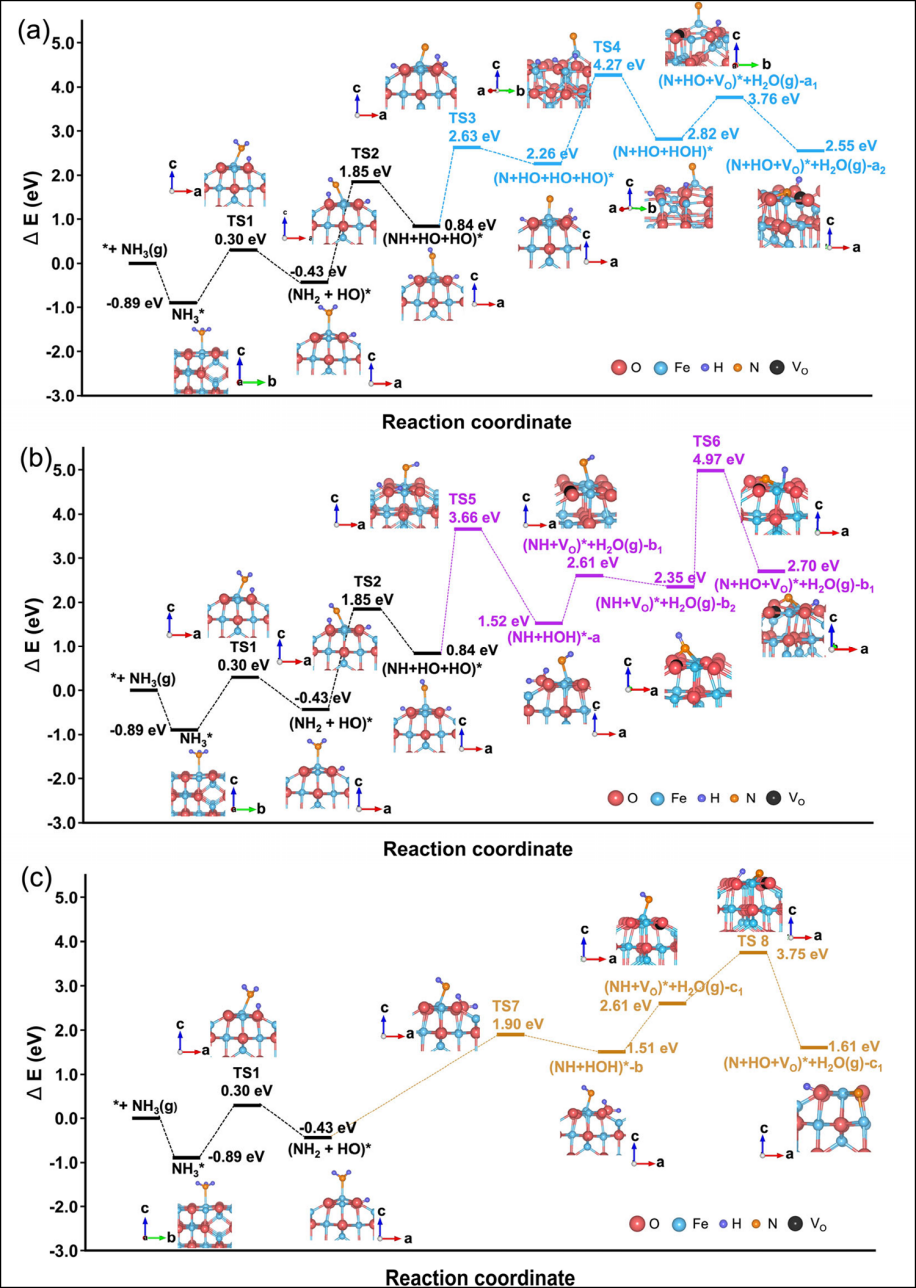
Potential energy diagrams for the stepwise reduction of Fe_3_O_4_(001) by NH_3_. The energy profiles depict three reaction pathways for NH_3_ oxidation dehydrogenation: (a) first pathway, (b) second pathway, and (c) third pathway. Each curve illustrates the relative energetics of adsorbed intermediates and transition states along the corresponding reaction route. Δ*E* values are referenced to the total energy of the pristine Fe_3_O_4_(001) surface plus gas-phase NH_3_(denoted NH_3_(g)). Crystallographic directions of *a*, *b*, and *c* correspond to [110], [1̅10], and [001], respectively.

The second dehydrogenation step begins with the NH_2_ fragment tilting along the [1̅1̅0] direction, accompanied by the elongation and eventual cleavage of one N–H bond. The dissociated H atom binds to a nearby O atom on the surface, forming a second OH group. This step requires an activation barrier of 2.28 eV, higher than that of the first dehydrogenation event, reflecting the increased difficulty of removing H from the partially dehydrogenated NH_2_ species. Structural relaxation of the resulting (NH + OH + OH)* intermediate shows that the newly formed OH group rotates along the [1̅1̅0] direction to reach a more stable binding site.

To dissociate the final H from the NH fragment, we consider two possible reaction pathways. In Route 1 (blue pathway, Figure 2a), the NH group undergoes a third dehydrogenation step. This process involves a rotation of the NH moiety along the [110] direction, bringing the remaining H atom into proximity with a surface lattice O site. As the interaction between this O and the H strengthens and surpasses the residual N–H bond strength, the final N–H bond ruptures, with an activation barrier of 1.79 eV. This produces a third OH group, yielding the (N + OH + OH + OH)* intermediate. This dehydrogenation is endothermic by +1.42 eV, indicating that the final H abstraction is energetically costly.

Subsequent H migration between two neighboring OH groups forms a surface H_2_O species. This step, which involves proton transfer and reorganization of surface hydroxyls, proceeds with an activation barrier of 2.01 eV, making it one of the most energetically demanding steps. The process is also endothermic. The newly formed H_2_O molecule then desorbs from the surface, creating a surface V_O_. The desorption step requires an energy endothermicity of 0.94 eV, yielding the intermediate (N + OH + V_O_)* + H_2_O­(g)-a_1,_ where g represents the gas phase. The system subsequently relaxes to a more stable configuration, (N + OH + V_O_)* + H_2_O­(g)-a_2_, as the remaining N atom reconfigures into a tridentate geometry that coordinates to two surface Fe ions and one O ion. This rearrangement reduces the system energy and stabilizes the N-containing fragment on the surface. Overall, the complete reaction sequence—from the pristine Fe_3_O_4_(001) surface and gas-phase NH_3_ (* + NH_3_(g)) to the final configuration (N + HO + V_O_)* + H_2_O­(g)-a_2_—is endothermic by 2.55 eV. Among all elementary steps, H migration leading to H_2_O formation is the rate-determining step owing to its high activation barrier.

In the alternative reaction pathway, Route 2 (purple pathway in Figure 2b), the (NH + OH + OH)* intermediate undergoes a H migration process forming a surface H_2_O molecule. This proton-transfer step proceeds with an activation barrier of 2.82 eV, making it the rate-limiting step of surface-bound water formation in Route 2. The newly formed H_2_O molecule tilts along the [1̅1̅0] direction and lifts slightly from the surface, yielding the (NH + –H_2_O)*-a configuration. Subsequent H_2_O desorption from the surface requires an additional energy of 1.09 eV, leaving behind an V_O_ on the surface, producing the intermediate (NH + V_O_)* + H_2_O­(g)-b_1._ The system then relaxes into a more stable configuration, (NH + V_O_)* + H_2_O­(g)-b_2_, as the N binds strongly to the surface via two N–Fe bonds and one N–O bond. This tridentate coordination closely resembles that obtained in Route 1, indicating that both pathways ultimately converge to a similar N-anchored final state. Then the system transitions to the ultimate state (N + HO + V_O_)* + H_2_O­(g)-b_2_ by dissociation of the third H atom, which requires overcoming a barrier of 2.62 eV. The overall reaction along Route 2 is endothermic by 2.70 eV, slightly higher than the total energy cost of Route 1. As in Route 1, H migration leading to H_2_O formation remains the rate-determining step, underscoring the central role of proton mobility in governing NH_3_ dehydrogenation and vacancy formation on the Fe_3_O_4_(001) surface.

The third dehydrogenation pathway, represented by the brown lines in Figure 2c, proceeds from the (NH_2_–OH)* intermediate and involves direct interaction between the NH_2_ fragment and an existing surface OH. In this route, the NH_2_ species rotates along the [110] direction and approaches a nearby surface lattice O ion. As the N–H bond stretches and weakens, it eventually cleaves, and the released H atom binds to the adjacent OH. This step produces a surface-bound H_2_O molecule and requires an activation energy of 2.33 eV. The newly formed H_2_O then lifts along the [001] direction into a stable configuration, denoted as (NH–H_2_O)*-b.

The next step is desorption of the H_2_O molecule from the surface, which requires 1.1 eV and yields the intermediate configuration (NH + V_O_)* + H_2_O­(g)-c_1_. Following H_2_O desorption, the system undergoes a third H dissociation event to reach its final configuration, (N + HO + V_O_)* + H_2_O­(g)-c_1_. This final H-removal step has an activation barrier that requires 1.14 eV. Notably, in the final state, the remaining N atom directly occupies the V_O_ created by H_2_O desorption, effectively substituting into the lattice site. This substitutional incorporation contrasts with Routes 1 and 2, where the N fragment remains adsorbed on the surface, coordinated to two Fe ions and one O ion. Among the three pathways, Route 3 emerges as the most favorable both thermodynamically and kinetically. Its advantage stems from avoiding the long-range H migration required in the other mechanisms, enabling a more direct and efficient progression toward H_2_O formation, vacancy creation, and N incorporation.

The calculations above reveal that the conventional route involving proton diffusion and isolated V_O_ formation is energetically unfavorable. Instead, we identify a more efficient “cooperative” mechanism: when multiple H fragments from NH_3_ dissociation cluster at a single lattice oxygen site, the barrier for H_2_O formation and desorption is lowered. This localized reduction creates a transiently active site that is immediately accessible for N incorporation. This coupled sequence—where H_2_O removal and N filling are energetically linked—represents the most favorable channel for surface transformation.

### 3.3 Nitride and N_2_ Formation

The reduction of Fe_3_O_4_(001) by NH_3_ induces a dynamic restructuring of the surface and subsurface layers, driven by the formation and migration of V_O_. A critical consideration of this process is the dynamic interplay between V_O_ and N species, which determines whether the system undergoes self-limiting surface passivation or sustained deep nitridation. To address this, we investigate the vacancy-coupled redistribution of N, as direct N–O exchange between layers is energetically unfavorable due to the high cost of simultaneous bond breaking.

As illustrated by the blue pathway in Figure 3a, starting from the intermediate (N + HO + V_O_)* + H_2_O­(g)-a_2_ configuration, the surface V_O_ preferentially migrates into the subsurface. This vacancy relocation is equivalent to the upward diffusion of a neighboring O ion to the surface, producing the (N + HO + V_O_)* + H_2_O­(g)-a_3_ state. Throughout this exchange, the surface N species preserves its stable tridentate coordination. While this vacancy exchange involves a high activation barrier of 2.43 eV and is endothermic by 0.89 eV, it establishes a critical kinetic channel for N to penetrate the subsurface. By facilitating the inward flux of vacancies and the corresponding outward flux of lattice oxygen, this mechanism prevents terminal site-blocking and enables the progressive nitridation of the Fe_3_O_4_ lattice.

**Figure 3:**
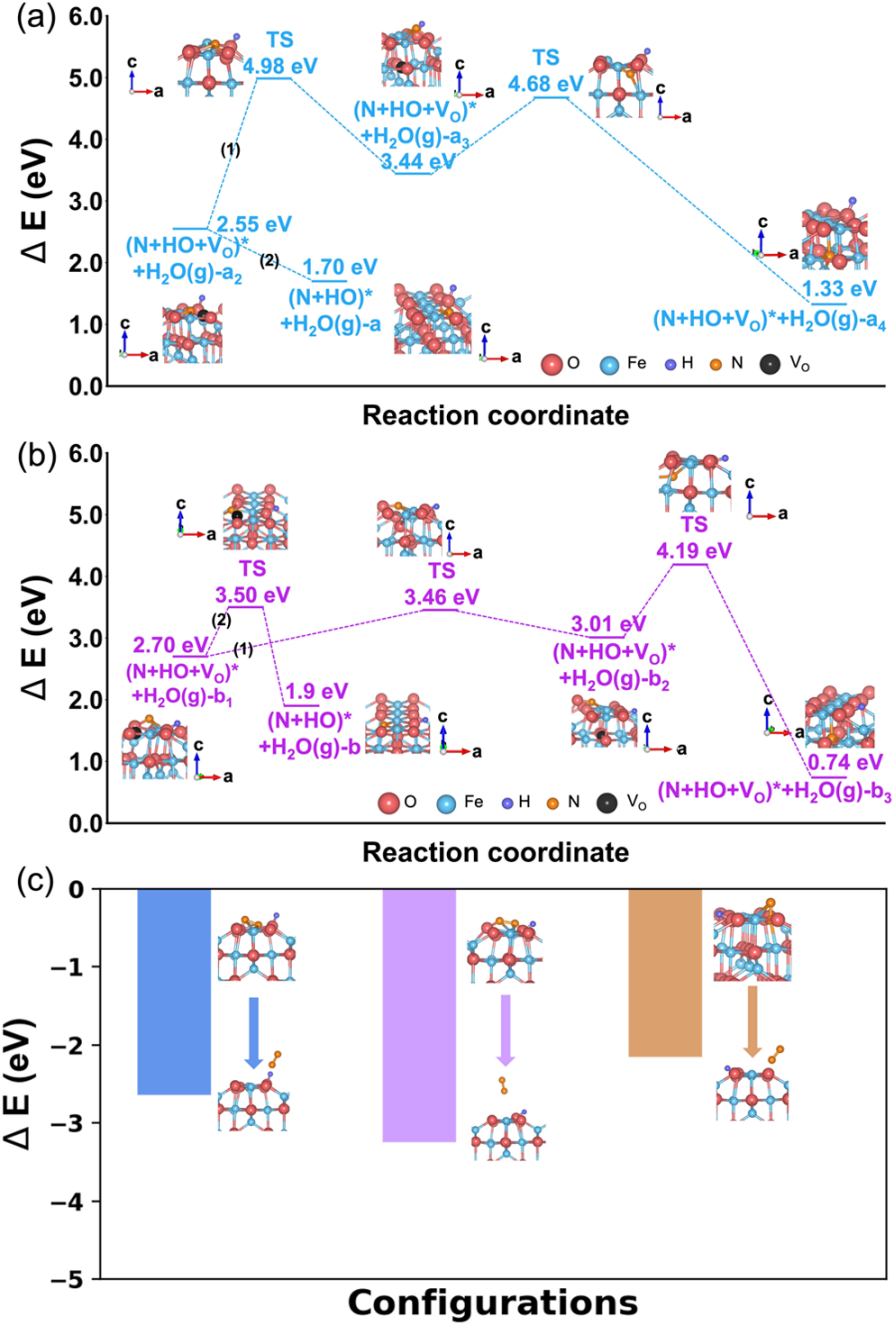
Thermodynamic and kinetic landscapes for nitridation and N_2_ recombination. (a, b) Potential energy diagrams illustrating the nitridation pathways during NH_3_-mediated reduction of Fe_3_O_4_(001). Energy changes (Δ*E*) are referenced to the combined energy of the pristine Fe_3_O_4_(001) surface and a gas-phase NH_3_ molecule. The landscapes compare two competing mechanisms: Path 1: Sequential subsurface V_O_ migration followed by N incorporation into the lattice. Path 2: Direct on-surface V_O_ filling by adsorbed N. The profiles identify transition states and activation barriers, highlighting the thermodynamic driving force for Fe–N bond formation in an O-deficient lattice. (c) Potential energy diagram for molecular N_2_ formation. Δ*E* values are referenced to a surface state containing one adsorbed N atom—corresponding to the (N + HO + V_O_)* + H_2_O­(g)-a_2_/b_1_/c_1_ configurations in Figure 2—plus half the energy of a gas-phase N_2_ molecule. All investigated pathways converge toward spontaneous N_2_ evolution with net energy release, highlighting the intrinsic thermodynamic favorability of N_2_ desorption under reducing conditions.

Extensive experimental studies have identified Fe nitride formation as a hallmark of NH_3_-based reduction,^25,26,35^ reflecting a strong thermodynamic preference for N incorporation into O-deficient iron-oxide lattices. In the present system, the surface-bound N exhibits a pronounced tendency to migrate into the subsurface regions, where it preferentially occupies V_O_ sites. Beginning from the (N + HH + V_O_)* + H_2_O­(g)-a_3_ configuration, N undergoes a coordinated migration process that involves breaking its three surface bonds (two Fe–N and one O–N bond). The N atom then diffuses into the vacant lattice site beneath the surface, yielding the fully incorporated (N + HO + V_O_)* + H_2_O­(g)-a_4_ configuration. In this final state, N forms four N–Fe bonds in a tetrahedral geometry that closely resembles the coordination environment found in bulk Fe nitride phases. Notably, this subsurface incorporation requires an activation barrier of 1.24 eV yet is strongly exothermic, releasing 2.11 eV of energy. The significant energy gain highlights the substantial thermodynamic driving force for nitride formation, driven by the greater stability of N–Fe bonding in the lattice compared to the surface-bound configurations. This pathway therefore provides a clear mechanistic basis for the experimentally observed enrichment of N within NH_3_-reduced Fe oxides.^25–27^

The purple pathway in Figure 3b further illustrates the dynamic evolution of V_O_ on the NH_3_-reduced Fe_3_O_4_(001) surface. Starting from the (N + HO + V_O_)* + H_2_O­(g)-b_1_ configuration, the surface V_O_ migrates into a neighboring subsurface layer, producing the b_2_ intermediate. This vacancy exchange proceeds through a modest activation barrier of 0.76 eV and is only mildly endothermic (+0.31 eV). Once the vacancy settles in the subsurface, a more substantial structural reorganization follows: the surface-bound N atom—initially coordinated to two Fe and one O—diffuses into the newly formed subsurface V_O_ site. This incorporation step yields the b_3_ configuration and requires an activation barrier of 1.18 eV, but is strongly exothermic (−2.27 eV), reflecting the substantial thermodynamic stabilization associated with forming a tetrahedrally coordinated N–Fe environment characteristic of nitride phases.

Comparison of the full reaction pathways further highlights the energetic preference for N incorporation into the lattice. Subsurface N migration in Route a is exothermic by −1.22 eV, whereas Route b yields an even larger stabilization of −1.96 eV. These results clearly demonstrate the inherent driving force for N to occupy O-deficient lattice sites, consistent with experimental evidence for nitride formation during NH_3_-based reduction.

A critical consideration in the nitridation of Fe_3_O_4_(001) is whether the process is inherently self-limiting. If on-surface N species (N_surf_) were to exclusively occupy surface V_O_, the resulting N-terminated surface would eventually passivate, halting further NH_3_ activation. To evaluate the feasibility of sustained catalysis, we investigate the competition between surface V_O_ filling and the migration of V_O_ into the subsurface. As illustrated in Figure 3a, N_surf_ can occupy a surface V_O_ without a kinetic barrier (Path 2). However, the resulting (N + HO)* + H_2_O­(g)-a configuration is energetically less favorable than the state reached via Path 1, (N + HO + V_O_)* + H_2_O­(g)-a_4_. In the latter, the surface V_O_ first migrates into the subsurface, followed by the migration of N_surf_ into the newly created subsurface vacancy site. This thermodynamic instability suggests that immediate surface-level N occupancy is likely a transient state rather than a terminal one.

Further analysis in Figure 3b reveals a distinct kinetic competition between two pathways: (1) Subsurface O migration to a surface V_O_ (O_sub_ → V_O, surf_) with a barrier of 0.76 eV (Path 1); (2) On-surface N diffusion into the same surface V_O_ (N_surf_ → V_O, surf_) with a barrier of 0.8 eV (Path 2). This kinetic proximity of these two barriers suggests that both processes are operative under reaction conditions.

Ultimately, V_O_ migration into the subsurface is the key step that sustains catalytic turnover. By relocating V_O_ to the second or third atomic layers, the system creates a pathway for deep nitridation while simultaneously regenerating the surface O sites required for continuous H_2_O formation and removal. This mechanism is consistent with previous observations in selective redox catalysis, where the outward diffusion of lattice O and the corresponding inward flux of vacancies prevent surface site-blocking and maintain steady-state activity.^57–59^ Together, these atomic-scale insights reveal how vacancy mobility and N incorporation synergistically reshape Fe_3_O_4_ during reduction, and they suggest that controlling the generation and diffusion of oxygen vacancies may offer a strategy to modulate the depth and uniformity of nitridation in industrial NH_3_-based oxide reduction.

Experimentally, N_2_ evolution is also frequently observed as a major gaseous product during NH_3_-mediated reduction of iron oxides,^26,35^ indicating that surface N species may recombine rather than incorporate. To assess the feasibility of N_2_ formation, we employ a computationally efficient model in which a second N atom is introduced near a surface-bound N atom in the (N + HO + V_O_)* + H_2_O­(g)-a_2_/b_1_/c_1_ configurations (previously shown in Figure 2). This setup enables systematic evaluation of three distinct N_2_ formation pathways. The relative energy change (Δ*E*) for each route is calculated with respect to the energy of the system containing one adsorbed N atom plus half the energy of a gas-phase N_2_ molecule. Upon structural relaxation, all three configurations spontaneously lead to N_2_ desorption from the surface, each accompanied by a net energy decrease, as shown in Figure 3b. While simplified, this model captures the essential thermodynamics of N_2_ evolution and reveals that N_2_ desorption is energetically favorable whenever two reactive N species coexist on the surface. These insights clarify the competition between nitridation (N incorporation into vacancies) and denitridation (N removal as N_2_)—two processes that govern the phase composition, defect chemistry, and reduction efficiency of Fe_3_O_4_ under NH_3_ atmospheres.

The results presented above provide atomistic insights into how NH_3_ functions as an effective reductant for Fe oxides, offering a viable pathway for sustainable iron production while addressing a major challenge associated with H_2_-based direct reduction—namely, the difficulty of storing and transporting molecular H_2_. By elucidating the detailed adsorption and dehydrogenation mechanisms of NH_3_ on Fe_3_O_4_(001), we identify the key kinetic bottlenecks and thermodynamic driving forces that govern the overall reduction process. A central finding is that H migration is the rate-limiting step in forming and desorbing H_2_O, which in turn determines the rate at which O vacancies first emerge on the surface. Once formed, these vacancies play a decisive role in steering the reaction pathway: they facilitate N incorporation into the Fe oxide lattice, enabling the formation of stable Fe–N bonds and ultimately Fe nitride phases. At the same time, the system also supports N_2_ evolution, a thermodynamically downhill pathway that prevents excessive N buildup. This dual outcome—nitride formation and N_2_ release—mirrors experimental observations and reveals a self-regulating mechanism that balances N incorporation and removal.

Beyond the immediate relevance to ironmaking, these insights have broader implications for NH_3_-involved catalytic processes. Fe-based oxides and nitrides serve as catalysts or catalyst supports in reactions such as NH_3_ decomposition, selective catalytic reduction of NO_*x*_, and NH_3_ oxidation.^60–62^ Our identification of preferred NH_3_ adsorption geometries, dehydrogenation pathways, and the active role of surface and subsurface O vacancies provides fundamental insights into how Fe-based materials activate and transform NH_3_ under reaction conditions. In particular, mapping the energy barriers for N–H bond scission and H migration offers mechanistic guidance for tuning catalytic activity and selectivity, especially in processes where the formation, incorporation, or removal of N-containing intermediates directly influences performance. The demonstrated capability of N atoms to occupy lattice V_O_ sites and form tetrahedrally coordinated nitrides suggests strategies for designing nitride-based catalysts with enhanced stability and tailored electronic structure.

## 4 Conclusions

In summary, this work presents a DFT-based framework for NH_3_-mediated reduction on Fe_3_O_4_(001). NH_3_ adsorbs preferentially atop surface Fe sites in a vertical orientation, activates through lone-pair donation, and undergoes stepwise dehydrogenation leading to H_2_O formation and vacancy creation. The reduction sequence is endothermic overall, requiring external thermal energy—consistent with industrial high-temperature NH_3_ reduction processes. After H_2_O desorption, the system bifurcates into two dominant pathways: nitride formation, governed by vacancy or N atom migration, and N_2_ evolution, which emerges spontaneously and thermodynamically favors N removal. These atomistic insights advance the fundamental understanding of NH_3_-driven Fe oxide reduction and provide mechanistic guidance for designing more efficient NH_3_-based reduction technologies. They also inform the broader field of NH_3_ catalysis and green metallurgy, where controlling N incorporation and vacancy chemistry is essential for optimizing reactivity, selectivity, and material stability.
